# ReIU: an efficient preliminary framework for Alzheimer patients based on multi-model data

**DOI:** 10.3389/fpubh.2024.1449798

**Published:** 2025-01-03

**Authors:** Hao Jiang, Yishan Qian, Liqiang Zhang, Tao Jiang, Yonghang Tai

**Affiliations:** ^1^Engineering Research Center of Photoelectric Detection and Perception Technology, Yunnan Normal University, Kunming, China; ^2^Yunnan Key Laboratory of Optoelectronic Information Technology, Kunming, China; ^3^Department of Ophthalmology, Eye and ENT Hospital, Fudan University, Shanghai, China; ^4^Laboratory of Myopia, Chinese Academy of Medical Sciences, Shanghai, China; ^5^Shanghai Research Center of Ophthalmology and Optometry, Shanghai, China

**Keywords:** Alzheimer patients multimodal data, retinal vessel segmentation, biomarker extraction, preliminary patients screening, deep learning

## Abstract

The rising incidence of Alzheimer’s disease (AD) poses significant challenges to traditional diagnostic methods, which primarily rely on neuropsychological assessments and brain MRIs. The advent of deep learning in medical diagnosis opens new possibilities for early AD detection. In this study, we introduce retinal vessel segmentation methods based on U-Net ad iterative registration Learning (ReIU), which extract retinal vessel maps from OCT angiography (OCT-A) facilities. Our method achieved segmentation accuracies of 79.1% on the DRIVE dataset, 68.3% on the HRF dataset. Utilizing a multimodal dataset comprising both healthy and AD subjects, ReIU extracted vascular density from fundus images, facilitating primary AD screening with a classification accuracy of 79%. These results demonstrate ReIU’s substantial accuracy and its potential as an economical, non-invasive screening tool for Alzheimer’s disease. This study underscores the importance of integrating multi-modal data and deep learning techniques in advancing the early detection and management of Alzheimer’s disease.

## 1 Introduction

**Figure 1:**
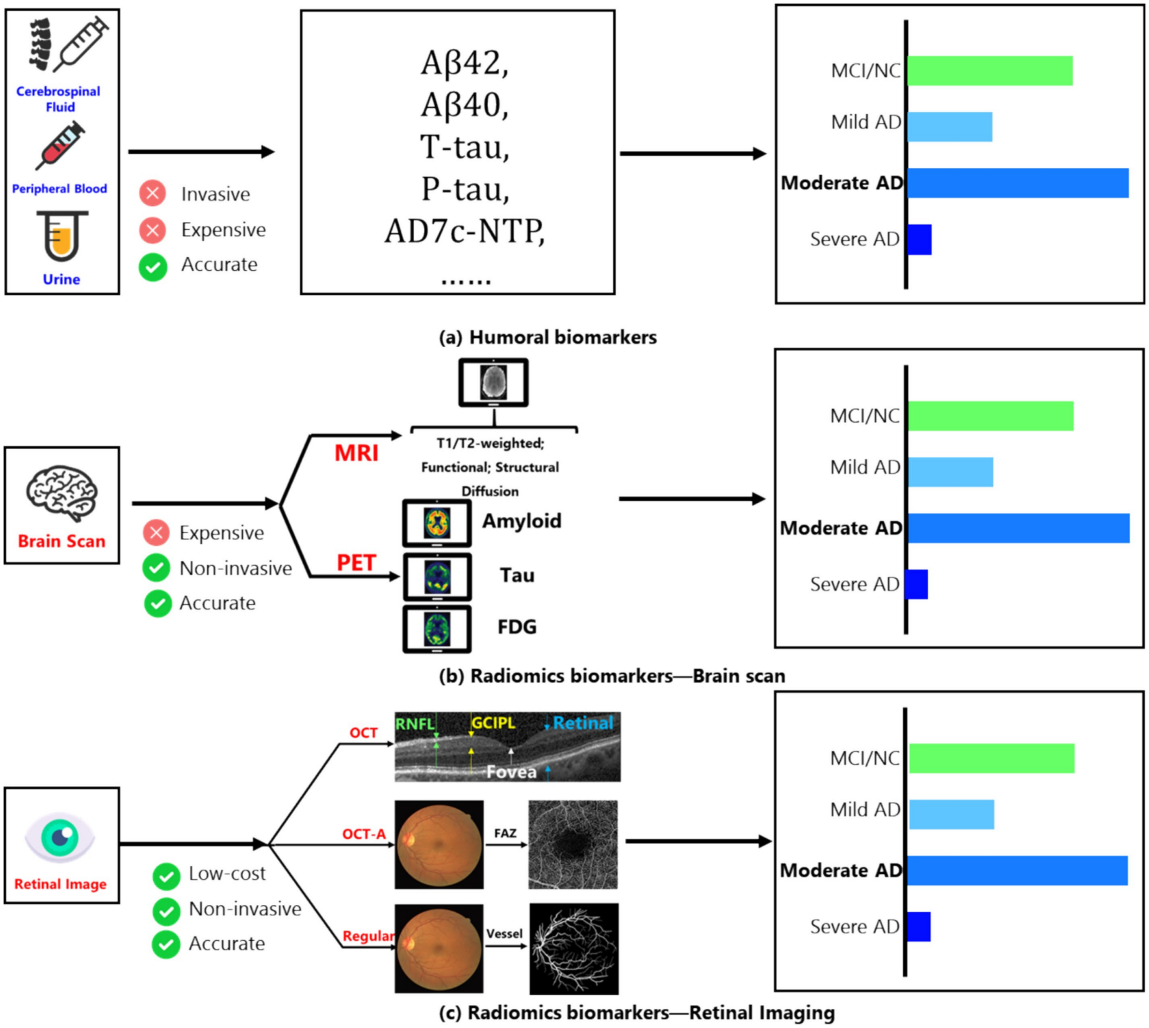
**(A)** Main humoral biomarkers for identifying the AD patients and normal people; **(B)** main brain scan biomarkers for identifying the AD patients and normal people; **(C)** main retinal image’s biomarkers for identifying the AD patients and normal people.

The World Alzheimer’s Disease Report 2023 illuminates a concerning statistic: approximately 75% of global dementia cases are undetected, with anticipated escalation to over 80 million by 2030 (1). This dire situation underscores the critical need for prompt diagnosis (2). However, the cause of Alzheimer’s Disease (AD) is still unclear, and this uncertainty in its development has made effective initial screening difficult for doctors. The key to finding useful preliminary screening methods is to identify significant biomarkers. Therefore, we first introduce the main biomarkers currently used to help diagnose AD: fluid biomarkers and imaging biomarkers in Figure 1 (3).

Among fluid biomarkers, cerebrospinal fluid (CSF) can directly reflect changes in brain tissue (4). Key CSF biomarkers related to AD include Aβ42, total tau protein (t-tau), and phosphorylated tau protein (p-tau). Aβ42 indicates the buildup of amyloid plaques in the cortex; t-tau reflects the degree of neurodegeneration; and p-tau is associated with neurofibrillary tangles. These core CSF biomarkers show high diagnostic accuracy during the mild cognitive impairment (MCI) stage, with sensitivity and specificity reaching 85–90%.

On the other hand, peripheral blood biomarkers are considered ideal for clinical trials due to their easier accessibility and lower invasiveness compared to CSF. Measuring specific components in peripheral blood (such as tau protein and Aβ levels) effectively distinguishes AD patients from healthy individuals (5). Additionally, the level of the AD7c-NTP protein in the urine of AD patients has been shown to correlate with neurofibrillary tangles, suggesting that urine could also serve as a biomarker for early AD diagnosis (6). While these fluid biomarkers are quite accurate, their collection can be expensive and invasive. Therefore, imaging-based biomarkers have emerged as a promising detection method, mainly involving fundus imaging and brain scans.

Among brain scanning methods, amyloid PET (7) provides information about the distribution and amount of amyloid plaques, showing strong agreement with post-mortem results and serving as the most direct diagnostic biomarker for Aβ changes in living subjects. FDG PET (7) measures glucose uptake in neurons and glial cells and is regarded as the most sensitive indicator of changes in synaptic function, with sensitivity up to 90%, effectively distinguishing AD from other forms of dementia. Furthermore, using fluorinated ligands for tau imaging has become a current focus of research due to its connection to clinical symptoms of AD.

MRI images (3) of the human brain are also important for discovering biomarkers. After stimulation, changes occur in neural activity, local blood flow, and regional oxygen consumption. Functional MRI (fMRI) can reflect abnormalities in neuronal and synaptic function through the relationship between blood flow changes and neural activity. Structural MRI (sMRI) measures the volume of specific brain regions or the whole brain, reflecting structural loss caused by cellular damage, axonal degeneration, and synaptic dysfunction. The earliest and most noticeable atrophy of the medial temporal lobe structures may be a key change occurring early in AD, with hippocampal atrophy being the best indicator of the progression from MCI to AD dementia. However, despite the minimally invasive nature of MRI/PET scanning technologies, their use is still limited due to high costs and equipment availability.

To reduce examination costs while keeping a low-invasiveness screening method, the retina, as a layered sensory tissue, has shown involvement in AD dementia in several studies (8). At the same time, retinal imaging technologies, such as optical coherence tomography (OCT) and OCT angiography (OCT-A), allow researchers to examine retinal structures (like the nerve fiber layer and ganglion cell layer) and small blood vessels (such as the avascular zone in the fovea) to investigate specific biomarkers in the retina (9, 10).

Research has identified several changes linked to the development of AD that may serve as potential biomarkers: (1) Changes in small retinal arteries and veins (11, 12); (2) Changes in blood vessel response in the fundus (13, 14); (3) Changes in the peripheral retina (15), among others. Moreover, with advances in AI technology, changes in retinal vessel segmentation have become an important area of research that combines AI and medicine. Researchers can now observe retinal blood vessel networks through OCT angiography (16); for example, a study (17) showed that the capillary network density in AD dementia patients is significantly lower compared to normal controls, with an increase in the area of the avascular zone in the fovea. Thus, using AI technology, automated precise segmentation of retinal vessels has become a low-cost, low-invasiveness method for detecting AD (18, 19).

In this context, we propose a high-precision automated method for retinal vascular segmentation, aligning the obtained capillary density with medical labels collected by specialists to explore the relationship between capillary density in fundus images and the severity of AD. Additionally, we utilized deep learning to build a preliminary classification network to assist doctors in diagnosis.

The remainder of the paper is organized as follows: Section 2 provides a mathematical description of the algorithm and dataset detail. Section 3 details the experimental setup, including evaluation benchmarks and compares several segmentation methods. Section 4 presents a discussion for further analysis. Finally, Section 5 concludes the paper.

## 2 Materials and methods

The algorithm presented in this paper consists of two main components. The first component involves vessel segmentation based on conventional fundus images. To ensure the accuracy of the vessel segmentation model, we employ a U-Net (20) architecture under supervised learning as Component One, and an Iterative Multi-Modal Registration and Learning (IRL) approach (21) under weakly supervised learning as Component Two. The rationale behind this choice is that the supervised learning model can capture the primary structure of retinal vessels, while the semi-supervised learning approach, utilizing both fundus photography and fluorescein angiography images, can capture vessel features at multiple scales across different modalities. By integrating the features learned from both methods, we can ensure the accuracy of the derived retinal vessel density.

The second component focuses on a preliminary screening model based on the segmented vessel density. We first investigate the relationship between retinal vessel density and Alzheimer’s disease (AD). Subsequently, by incorporating collected medical indicators, we develop a preliminary screening model using deep learning techniques to assist physicians in diagnosing AD. Fundus vasculature segmentation was conducted using Tesla V100*2 hardware, while the classification task was executed on an RTX 3060. The algorithm was implemented in Python. Besides the main technical terminology’s abbreviation used in this paper was listed as below: (1) Alzheimer Disease (AD); (2) Retinal Nerve Fiber Layer (RNFL); (3) Ganglion Cell Inner Plexiform Layer (GCIPL); (4) Foveal Avascular Zone (FAZ); (5) Retinal Vessel Density (RVD); (6) Fundus Photography (FP); (7) Fluorescein Angiography (FA); (8) Mini-mental State Examination (MMSE). The Mini-Mental State Examination (MMSE) is a widely used cognitive screening tool that assesses cognitive function and helps in the detection of cognitive impairment, including Alzheimer’s disease. It consists of a 30-point questionnaire evaluating areas such as orientation, memory, attention, language, and visuospatial skills, with lower scores indicating greater cognitive impairment. The MMSE is commonly used in both clinical and research settings to monitor cognitive changes over time. We used ChatGPT-4o (OpenAI, version May 2024) to assist with language polishing and refinement of the manuscript content.

### 2.1 Dataset

#### 2.1.1 DRIVE dataset

The Digital Retinal Images for Vessel Extraction (DRIVE) (22) dataset is widely used for retinal vessel segmentation tasks. It contains 40 color fundus images in JPEG format, including 7 cases with abnormal pathologies. These images were collected as part of a diabetic retinopathy screening program in the Netherlands and were captured using a Canon CR5 non-mydriatic 3CCD camera with a 45-degree field of view (FOV). Each image has a resolution of 584 × 565 pixels and is represented with eight bits per channel across three color channels. In this paper, DRIVE was used to pre-training both IRL and U-Net.

#### 2.1.2 HRF dataset

The High-Resolution Fundus (HRF) (23) dataset is designed for retinal vessel segmentation and consists of 45 high-resolution fundus images, organized into 15 subsets. Each subset includes one image from a healthy subject, one image from a patient with diabetic retinopathy, and one image from a patient with glaucoma. The images have a resolution of 3,304 × 2,336 pixels, and the dataset is divided into 22 training images and 23 testing images. In this paper, HRF was used for evaluation.

#### 2.1.3 PRIME dataset

The PRIME-FP20 (21) dataset comprises 15 pairs of ultra-widefield (UWF) color fundus photographs (FP) and fluorescein angiography (FA) images, captured concurrently from baseline images of patients enrolled in the PRIME study. The images were acquired using Optos California and 200Tx cameras, with each image having a resolution of 4,000 × 4,000 pixels. All images are stored in 8-bit TIFF format with lossless LZW compression. In this study, the PRIME-FP20 dataset is used to help the model learn macrovascular features. However, due to the limited size of the PRIME-FP20 dataset and the lack of pre-registration, directly using FP images for prediction results in vessel maps with noticeable misalignment compared to the ground truth labels.

#### 2.1.4 Collected dataset

This study collaborated with the Biomedical Engineering Research Institute of Peking Union Medical College to collect eye images and corresponding medical indicators from 400 Alzheimer’s disease (AD) patients and 400 healthy individuals. The conventional fundus imaging was performed using Canon equipment, while the ultra-widefield fundus imaging utilized devices from Optos, but only conventional retinal images are aligned with clinical data.

### 2.2 Vessel segmentation

The vessel segmentation used in this paper includes three parts: (1) Supervised Learning by U-Net; (2) Weakly supervised Learning by IRL; (3) Multi-scale vessel information fusion by a general image fusion framework based on convolutional neural network (IFCNN) (24), which had been depicted in Figure 1.

U-Net, initially trained on standard fundus images (DRIVE/HRF) and fine-tuning with PRIME-FP20 (21), enhancing its segmentation capability for fundus images in different scales. The procedure of fine-tuning in this paper can be denoted as Equation 1. The $\Omega \left(\omega \right)$represents the loss function of the final U-Net, L2FT means the L2-Normalization operation, ω and $\omega_{\mathrm{Pretrained}}$ denotes the parameters of fine-tuning and pre-trained architecture separately:


(1) $$\Omega \left(\omega \right)=L2FT\omega -{\omega_{\mathrm{Pretrained}}}_{2}^{2}$$

Due to the imaging techniques used for ultra-widefield fundus images, obtaining precise vascular segmentation from these images is quite challenging. As a result, IRL does not employ traditional supervised learning to train models for segmenting ultra-widefield fundus images. Instead, IRL first utilizes a pre-trained segmentation model for fundus vessels, such as U-Net, and subsequently performs multimodal registration using concurrently collected FP and FA images. Weakly supervised learning is then introduced to optimize the model, enabling it to adapt to vascular segmentation across various scales and imaging methods.

For each iteration t, IRL extract the vessel map from the FA image $Y_{FA}^{i,t}$ and align it with the FP vessel coordinates $Q_{a}^{i,t}$ using a transformation $T_{B}$ optimized to minimize the Chamfer distance. This produces a registered FA vessel map, which is used as a pseudo-label for weak supervision. Equations 2–5 collectively explain how the is implemented. The specific details are as follows:


(2) $$T_{B}\left(Y_{FA}^{i,t}\right)\approx Q_{a}^{i,t}$$

Then, the registered FA vessel map $Y_{a-c}^{i,t}$ serves as a pseudo-label to guide model training. The IRL update the model weights $W^{t+1}$ by minimizing the loss function over the pseudo-labeled data $\left(X_{c}^{i},Y_{a-c}^{i,t}\right)$. The L measures the difference between model predictions and the pseudo-labels. After each iteration, we compute the loss and update the pseudo-labels improving the segmentation with each cycle:


(3) $$W^{t+1}=\arg\min_{W}\overset{M}{\underset{i=1}{\sum }}L\left(f\left(X_{c}^{i},Y_{a-c}^{i,t}\right),Y_{a-c}^{i,t}\right)$$

**Figure 2:**
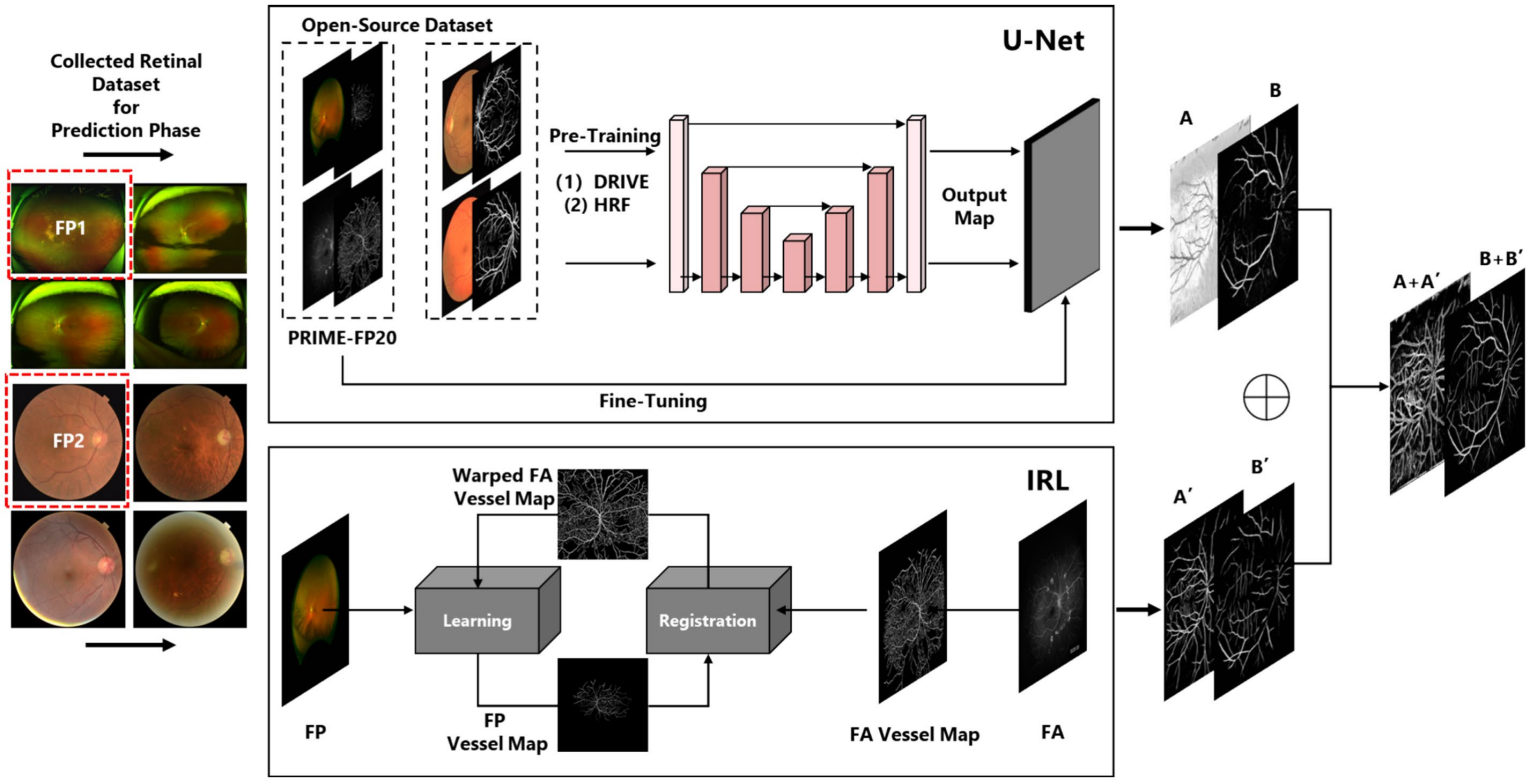
The detailed vessel segmentation procedure of ReIU.

Therefore, following (24), we fused the vessel maps and reapplied binarization, outlining this workflow in the Figure 2. The fusion framework was pre-trained by a medical image dataset:


(4) $$Loss_{CNN}\left(\omega \right)=FT\omega_{\alpha }-{\omega_{\mathrm{IF}}}_{2}^{2}$$

(5) $$y_{\mathrm{pred}}=F\left\{f_{CNN}\left(P_{IRL},P_{FT}\right)+c\left(N\right)\right\}$$

The $Loss_{CNN}\left(\omega \right)$represents the loss function of fusion architecture, FT which means the L2-Normalization operation, $\omega_{\alpha }$ denotes the parameters of fine-tuning, and $\omega_{\mathrm{IF}}$ means the pre-trained CNN. Besides, $y_{\mathrm{pred}}$ means the obtained vessel map. $P_{IRL}$ and $P_{FT}$ denote the first segmentation results of iteration registration learning and Fine-tuning U-Net, respectively. $f_{CNN}$ means the image fusion procedure of two vessel maps, $c\left(N\right)$ denotes the noise of images. And F means the binarization operation of the fusion result. The visualized introduction to iteration registration learning and fusion procedure in this paper was shown in Figure 2.

### 2.3 Patient preliminary screening

Following the initial vascular segmentation procedures, blood vessel maps were obtained for two cohorts comprising 400 Alzheimer’s Disease (AD) patients and 400 healthy individuals, totaling 800 participants. Initially, we developed a multimodal dataset comprising 400 Alzheimer’s disease (AD) patients and 400 healthy controls, integrating fundus images with corresponding medical textual data. However, considering the potential impact of diabetes on fundus imaging accuracy, we excluded individuals with obesity (a high-risk group for diabetes) based on BMI criteria. The refined dataset is detailed in first table. This dataset encompasses various demographic and clinical parameters, including Gender (M for male, F for female), Age, Body Mass Index (BMI), Systolic Blood Pressure (SBP), Diastolic Blood Pressure (DBP), Pulse Pressure (PD), Heart Rate (HR), and Education Level (coded as 1 for primary education, 2 for junior high, 3 for high school, 4 for undergraduate, and 5 for postgraduate levels). Additionally, the Mini-Mental State Examination (MMSE) scores are included, where a score below 23 denotes AD patients (Table 1).

**Table 1:** The statistical data of collected medical record of AD and control group.

Class	Patients (*n* = 379)	Control (*n* = 287)
Age	61.54 ± 5.06	66.51 ± 9.05
Sex(M/F)	186/193	145/142
BMI	22.50 ± 1.39	21.87 ± 1.69
SBP	146.49 ± 6.91	119.59 ± 5.72
DBP	89.72 ± 6.75	72.43 ± 6.87
PD	56.77 ± 8.81	47.16 ± 9.13
HR	66.88 ± 5.36	67.88 ± 7.51
EDU (1 ~ 5)	33/116/179/51/0	56/55/60/54/62
MMSE	19.92 ± 1.73	27.00 ± 1.98

**Figure 3:**
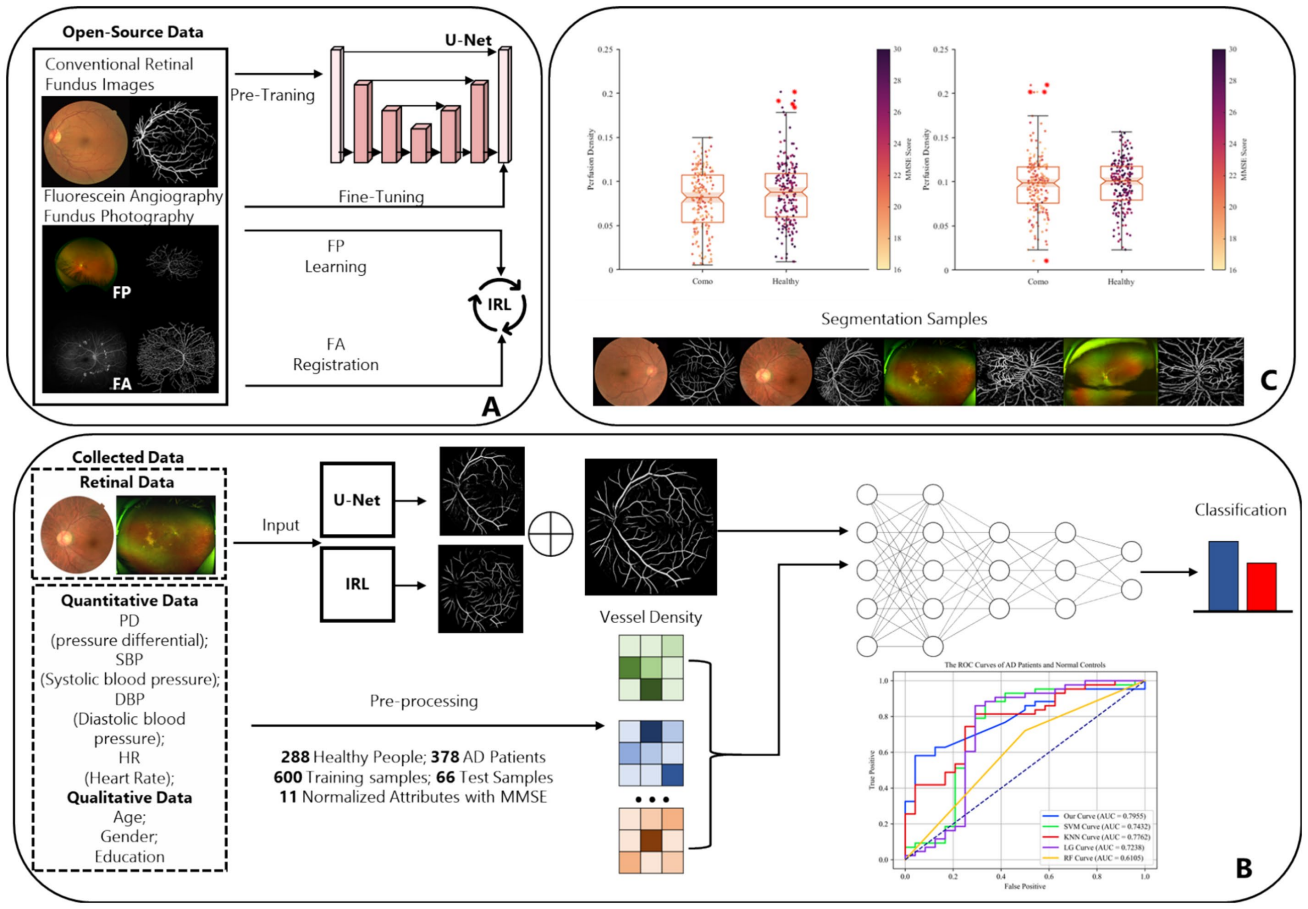
The complete design and experiment procedure of ReIU. **(A)** The workflow of the proposed segmentation part. **(B)** Data from 288 healthy individuals and 379 AD patients, processed through U-Net and IRL, then classified by a Dense-Net, as shown by ROC curves. **(C)** Box plots of MMSE scores by vessel density density for patients and healthy group, alongside segmented fundus image samples.

Then, we used fundus images from 379 cognitively impaired and 287 healthy individuals, classified by clinical features. A shallow dense net was designed for this small sample, achieving 79% accuracy in distinguishing AD patients. Within this framework, the retinal vessel density is incorporated as a feature extracted from the image, enhancing the final adjusted dataset for both training and testing purposes, as illustrated in Figure 3.

## 3 Results

To evaluate the effectiveness of our proposed framework, we conducted extensive experiments using open-source conventional retinal fundus images and collected multi-modality dataset. Our experiments are designed to assess the performance of each component in the pipeline, including the pre-trained and fine-tuned U-Net model, the iterative registration and weakly supervised learning (IRL) process, and the ReIU. We employed quantitative metrics and qualitative analysis to measure segmentation accuracy, robustness, ensuring a comprehensive evaluation of the model’s capability in real-world clinical settings. Besides, the extracted vessel density biomarker was valid by preliminary screening model on different data setting, which demonstrated that the vessel density is a potential solution to help doctor make non-invasive AD patient screening. First, we will introduce the dataset, we had used in this section.

### 3.1 Evaluate on mainstream retinal fundus datasets

**Figure 4:**
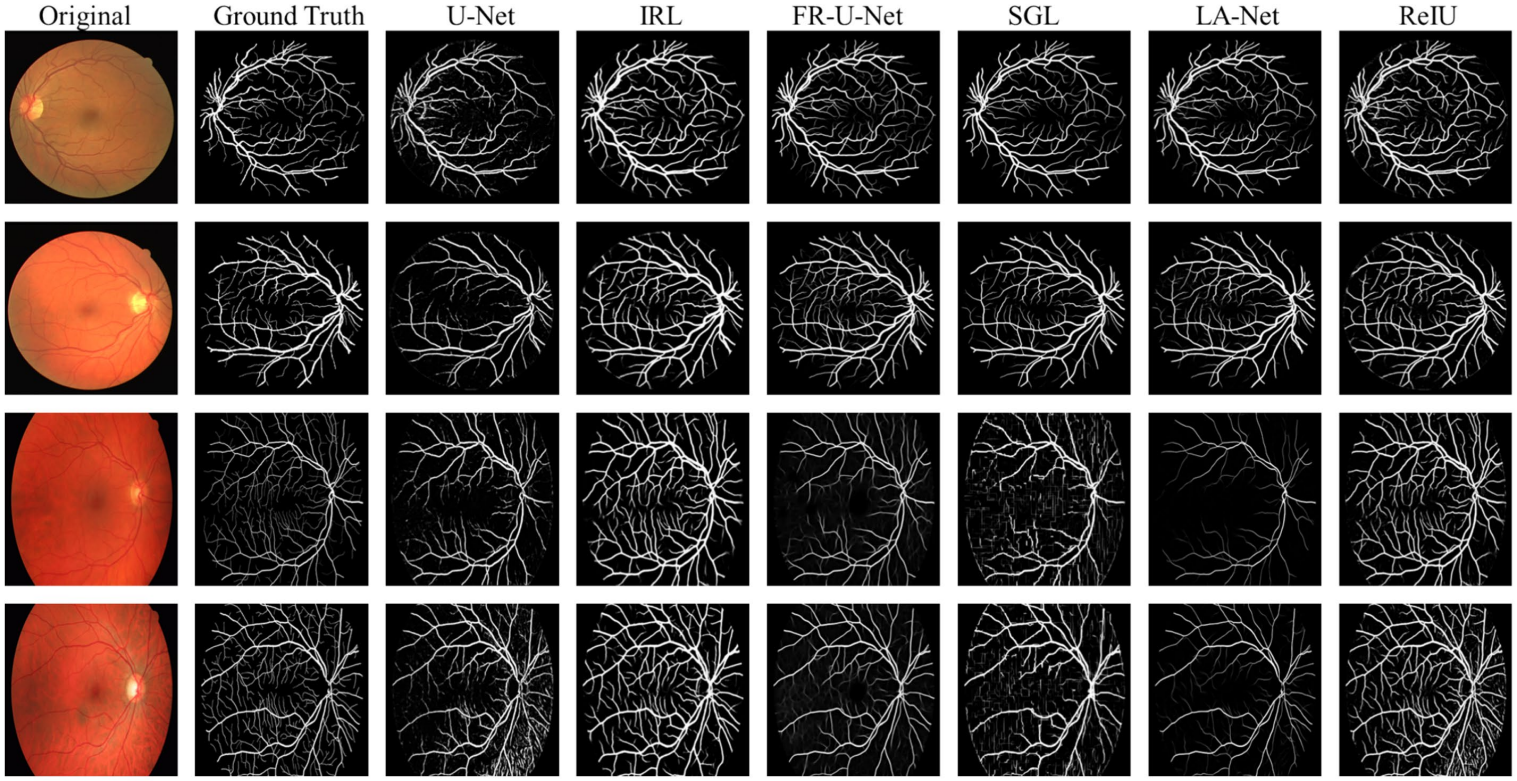
The segmentation samples of different mainstream methods on DRIVE and HRF dataset.

In the evaluation of conventional fundus images, segmentation outcomes using two standard datasets (DRIVE and HRF) were assessed across mainstream segmentation methods, as depicted in Figure 4. Our proposed method synergizes U-net with IRT, resulting in vascular diagrams that effectively integrate detail. Table 2 shows that ReIU achieved the highest Dice score (DRIVE: 0.791; HRF: 0.683). Despite potential retention of errors through fusion, ReIU maintained values close to the optimal zero on minimal indicators like volumetric overlap error (VOE) and relative volume difference (RVD). Although ReIU does not achieve the best results in terms of RVD, VOE, Precision, and Recall, its performance is still very close to that of mainstream models. These experiments confirm that ReIU satisfactorily extracts vessel density, maintaining requisite precision even comparing with mainstream vessel segmentation models.

**Table 2:** The proposed methods evaluated based on five main indicators.

DRIVE	U-Net (20)	IRL (21)	FR-U-Net (25)	SGL (26)	LA-Net (27)	ReIU
Dice↑	0.767	0.766	0.782	0.461	0.788	**0.791**
VOE↓	0.175	0.281	0.067	0.124	**0.006**	0.131
RVD↓	−0.154	0.321	0.074	−0.113	**−0.002**	0.144
Precision↑	**0.845**	0.670	0.803	0.491	0.827	0.741
Recall↑	0.709	**0.887**	0.860	0.437	0.823	0.845

HRF	U-Net (20)	IRL (21)	FR-U-Net (25)	SGL (26)	LA-Net (27)	ReIU
Dice↑	0.679	0.636	0.655	0.672	0.615	**0.683**
VOE↓	**0.041**	0.571	0.080	0.065	0.714	0.455
RVD↓	0.055	0.822	−0.070	**−0.053**	−0.524	0.603
Precision↑	0.668	0.498	0.788	0.729	**0.959**	0.559
Recall↑	0.698	**0.893**	0.726	0.686	0.455	0.886

The bold values in table indicate that the corresponding method achieves the best results on this dataset according to the specified evaluation metric.

The noticeably poorer performance of all methods on the HRF dataset compared to DRIVE is evident from the segmentation examples in Figure 4. Due to differences in imaging equipment, HRF images contain a higher number of red pixels, increasing segmentation difficulty and negatively impacting Dice accuracy across methods. Additionally, HRF images have a higher resolution (3,304 × 2,336) compared to DRIVE images (584 × 565), which may require more sophisticated models to achieve comparable performance.

The comparison model used in this paper is (1) U-Net (20); (2) IRL (21); (3) Full-resolution U-shape network (Fr-U-Net) (25); (4) Study group learning (SGL) (26); (5) multi-path networks based on U-Net for medical image segmentation (LA-Net) (27).

The Dice coefficient, ranging from 0 to 1, measures overlap between segmented and ground truth regions, with 1 indicating perfect overlap. Volumetric Overlap Error (VOE) also ranges from 0 to 1, where lower values denote better alignment, and 1 indicates no overlap. And Relative Volume Difference (RVD) represents the normalized volume difference and can range from negative infinity to positive infinity, with a value of 0 indicating identical volumes. Negative values show underestimation, while positive values reflect overestimation of the segmented volume compared to the ground truth. Finally, Precision and Recall both range from 0 to 1, where higher values represent more accurate segmentation. High values in Dice, Precision, and Recall suggest good segmentation, while low VOE and near-zero RVD indicate accurate volume matching. Equations for computing these metrics are provided in Equations 6–10, the comparison model are FR-U-Net (25), SGL (26), LA-U-Net (27):


(6) $$\mathrm{Dice}=2*\frac{\left|V_{\mathrm{Pred}}\cap V_{GT}\right|}{\left|V_{\mathrm{Pred}}|+|V_{GT}\right|}$$

(7) $$VOE=1-\frac{\left|V_{\mathrm{Pred}}\cap V_{GT}\right|}{\left|V_{\mathrm{Pred}}\cup V_{GT}\right|}$$

(8) $$RVD=\left(\frac{\left|V_{\mathrm{Pred}}|-|V_{GT}\right|}{\left|V_{GT}\right|}\right)$$

(9) $$\mathrm{Precision}=\frac{TP}{\left(TP+FP\right)}$$

(10) $$\mathrm{Recall}=\frac{TP}{\left(TP+FN\right)}$$

$V_{\mathrm{Pred}}$ denotes the obtained vessel map and $V_{GT}$ means the ground truth vessel map; TP, FP, FN represents the true positive, false positive, and True Negative, separately. The Dice coefficient is a maximization metric for segmentation accuracy, similar to Precision and Recall, while VOE and RVD are minimization metrics. Precision and Recall are particularly useful for identifying robust models in vessel segmentation. The segmentation samples are displayed in the Figure 4.

### 3.2 Preliminary screening on multi-model AD patient dataset

**Figure 5:**
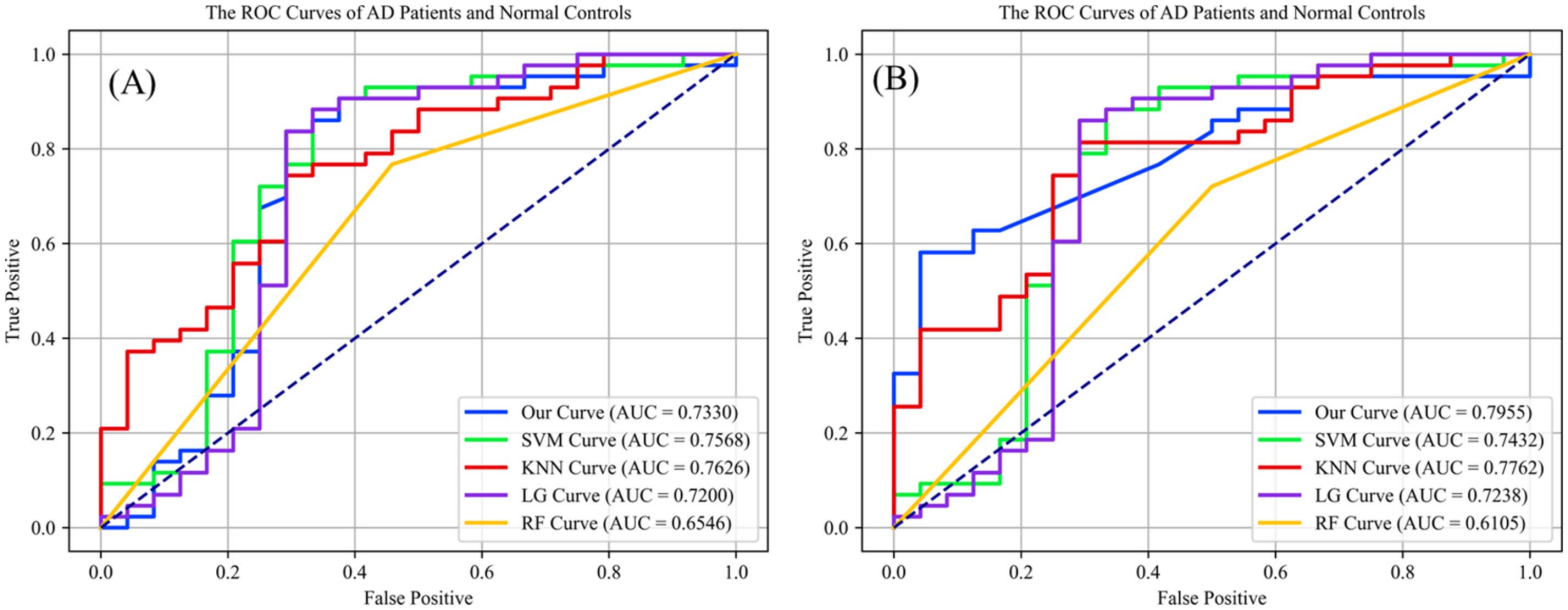
**(A)** The preliminary screening results without obtained vessel density on different classification methods, which the accuracy is 0.7320 on ReIU; **(B)** The preliminary screening results with obtained vessel density on different classification methods, which the accuracy is 0.7955 on ReIU.

This study assessed the Dense-Net’s detection capabilities and performance across various classification thresholds via the receiver operating characteristic (ROC) curve, plotting the false positive rate (FPR) against the true positive rate (TPR) obtained from classifier performance. Figure 5A illustrates the classification outcomes without vessel density biomarker, while Figure 5B includes biomarker. The diagonal line in a ROC curve serves as a visual reference, representing the baseline performance of random guessing. A step-like curve that moves away from the diagonal and towards the top-left corner indicates that the model’s accuracy improves with each additional training epoch.

Findings from Figure 5 indicate that Dense-Net achieved the highest accuracy, with vessel density providing significant optimization for the classification task. Notably, the ROC (Receiver Operating Characteristic) curve of a network model typically lies above the diagonal line, indicating the model’s performance correctness within the accuracy range. Here, a higher position on the curve signifies superior performance. The AUC curve is calculated using Equation 11, which is defined as follows:


(11) $$AUC=\frac{\sum_{\mathrm{insi}\in \mathrm{positiveclass}}\mathrm{ran}k_{\mathrm{insi}}-\frac{M^{*}\left(M+1\right)}{2}}{M^{*}N}$$

$\mathrm{ran}k_{\mathrm{insi}}$ represents the serial number of the first sample. (Probability scores are ranked from small to large), M and N are the numbers of positive and negative samples, respectively. And the sum formula only adds up the serial numbers of the positive samples. The experiment of a multi-model network can be divided into the segmentation of fundus images and text classification on the AD dataset.

Upon inclusion of vessel density in the classification algorithms, the second ROC curve elucidates a marked augmentation in discriminatory prowess, as evidenced by the ascension of the AUC values across most algorithms. The introduction of vessel density as a variable engenders a salient enhancement in the performance of shallow Dense-Net with its AUC surging from 0.7330 to 0.7955, thereby underscoring its substantial contribution to the algorithm’s diagnostic acumen.

Simultaneously, in the Figure 5 we can find that the support vector machine (SVM) algorithm demonstrates a modest increase in area under the curve (AUC), albeit less pronounced than the shallow Dense-Net model. This suggests that while SVM benefits from the incorporation of SCP density, its influence is not paramount. The K-nearest neighbors (KNN) and logistic regression (LG) algorithms show a marginal improvement in AUC, indicating a subtle enhancement in classification accuracy with the integration of vessel density.

Conversely, the random forest (RF) algorithm experiences a decrease in AUC when vessel density is included, suggesting a potential incongruity between this feature and the algorithm’s inherent classification mechanisms. This could indicate the presence of overfitting or an adverse interaction with the existing feature set within the Random Forest framework.

In summary, integrating vessel density into classification algorithms primarily improves the differentiation between AD patients and healthy controls, underscoring the diagnostic significance of this feature and advocating for its inclusion in predictive models for medical assessment. The introduced ReIU method, when combined with Dense-Net, assists clinicians in diagnosing AD using multi-source heterogeneous datasets. Its implementation notably enhances classification accuracy in clinical AD diagnosis by incorporating vessel density, thus validating the selection of vessel density as a biomarker for extraction.

## 4 Discussion

**Figure 6:**
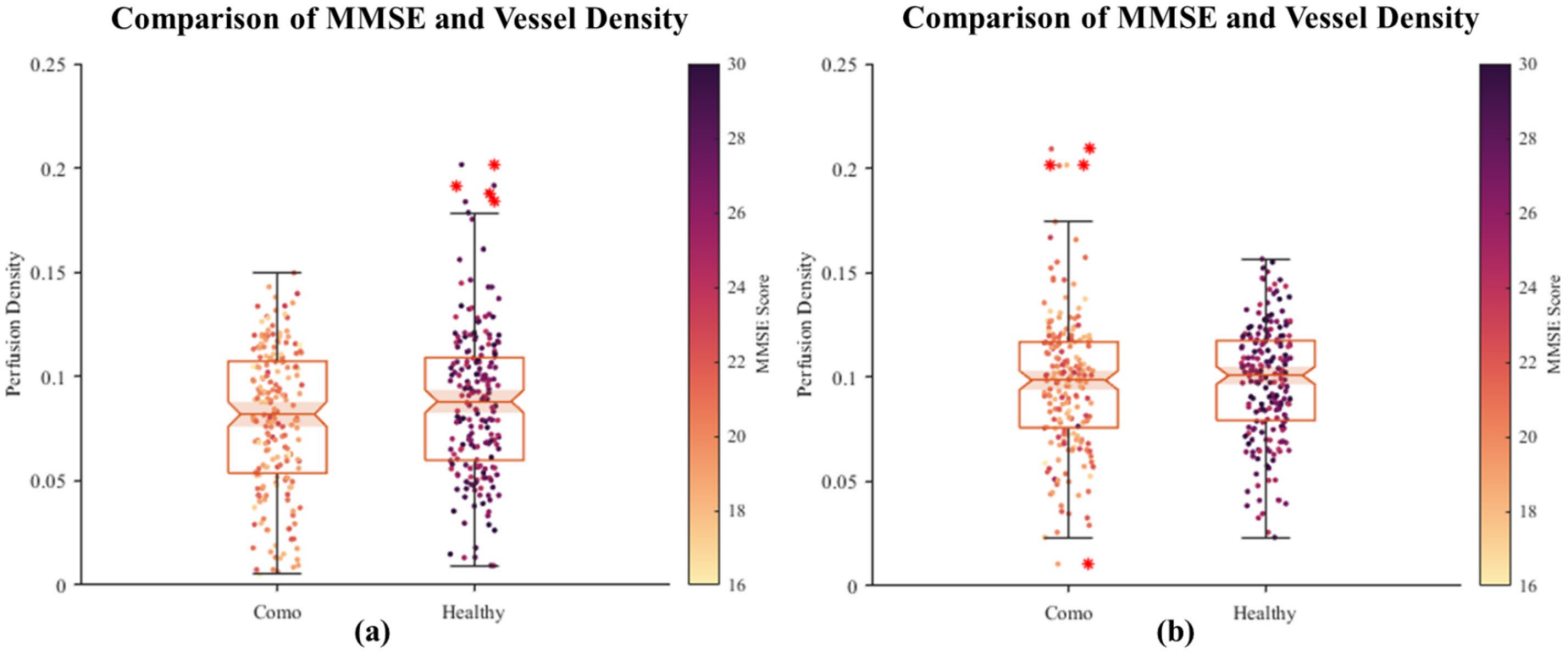
The scatter plot of correlation between fundus vascular density and MMSE. On the horizontal axis, the grouping distinguishes between healthy individuals and AD patients, while the vertical axis denotes the density of blood vessels in the fundus. Each scattered dot’s color intensity corresponds to the MMSE score, with darker shades indicating lower scores and lighter shades indicating higher scores. Based on the initial dataset of 400 healthy individuals and 400 patients, we randomly sampled two subsets, each consisting of 200 healthy individuals and 200 patients. Using their retinal images, we segmented and quantified the vascular density, visualized as two groups of images **(a, b)**. The results revealed no significant positive or negative correlation between retinal vascular density and MMSE scores.

In this section, we explore the relationship between retinal vessel density and the severity of Alzheimer’s Disease (AD). Although previous studies have reported significant differences in retinal vessel density between healthy controls and AD patients, retinal vessel density itself does not exhibit distinct pathological features that can effectively distinguish between healthy individuals and AD patients (28, 29). Therefore, in our experiment, we combined the vessel density data from healthy participants and AD patients to plot a scatter plot of MMSE scores against retinal vessel density. As shown in Figure 6, neither of the plots demonstrates a significant positive or negative correlation between MMSE scores and retinal vessel density.

**Figure 7:**
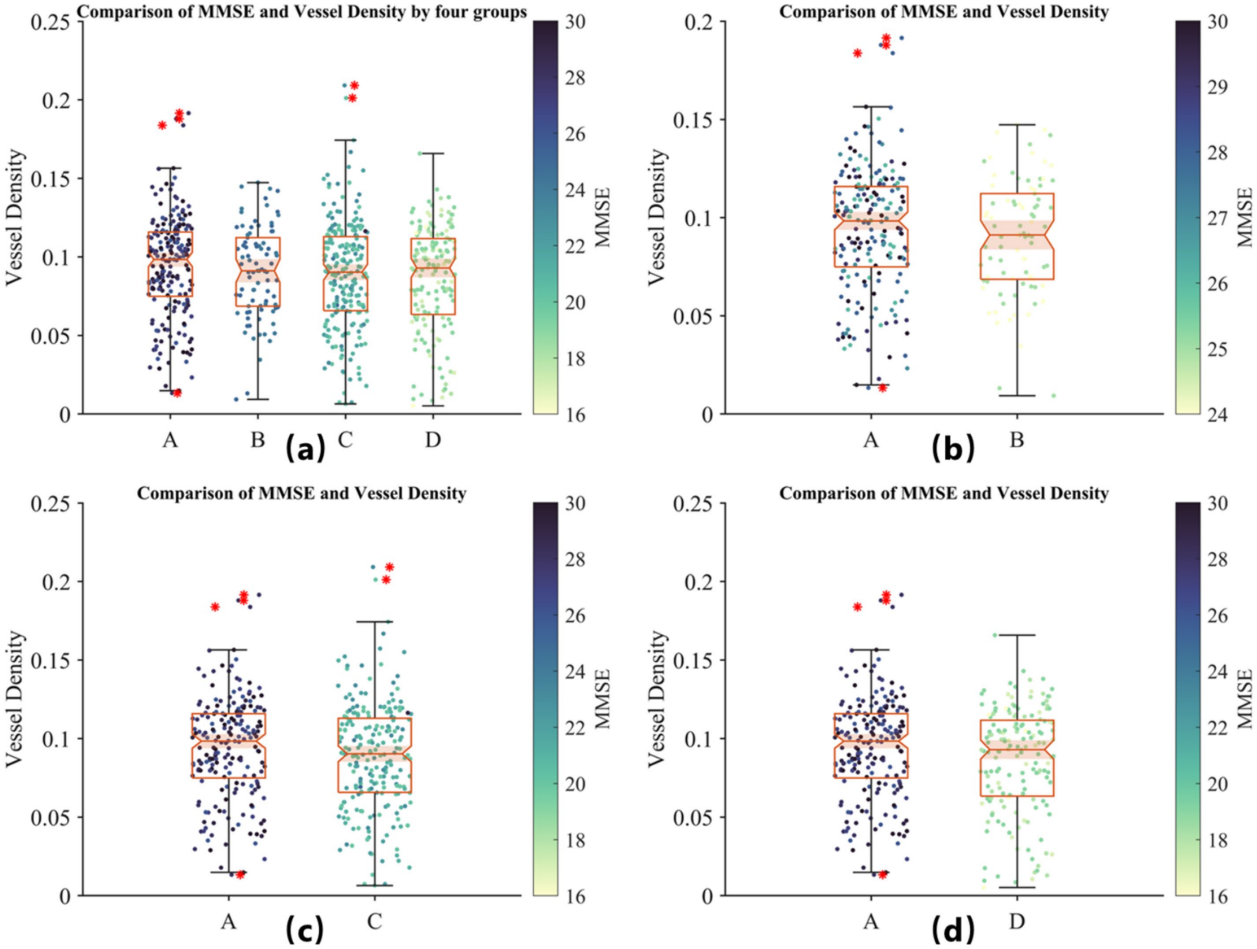
Comparison of MMSE scores and retinal vessel density across four groups based on AD severity. **(A)** Distribution of vessel density for all groups: Group A (MMSE >26, healthy controls), Group B (MMSE 20–25, mild AD), Group C (MMSE 10–19, moderate AD), and Group D (MMSE <9, severe AD). **(B)** Comparison of Group A and mild AD patients (Group B). **(C)** Comparison of Group A and moderate AD patients (Group C). **(D)** Comparison of Group A and severe AD patients (Group D). The color scale represents MMSE scores. No significant positive or negative correlation between MMSE scores and retinal vessel density is observed within groups, although vessel density tends to be lower in AD patients compared to healthy controls, suggesting its potential as a preliminary biomarker for AD screening.

Therefore, we conducted a further stratification based on MMSE scores to reflect the severity of AD. Group A represents individuals with MMSE scores greater than 26 (normal group); Group B includes patients with mild AD symptoms with MMSE scores between 20 and 25; Group C comprises patients with moderate AD symptoms with MMSE scores between 10 and 19; and Group D includes patients with severe AD symptoms with MMSE scores below 9. A scatter plot of MMSE scores against retinal vessel density for each group is shown in Figure 7.

In Figure 7, by observing the differences between Group A and the other groups, we find that although MMSE scores cannot reliably indicate the severity of AD, retinal vessel density does tend to be lower in the diseased groups compared to the healthy group. This suggests that retinal vessel density could serve as a potential biomarker for preliminary screening, although it should still be used in conjunction with a professional medical diagnosis.

**Figure 8:**
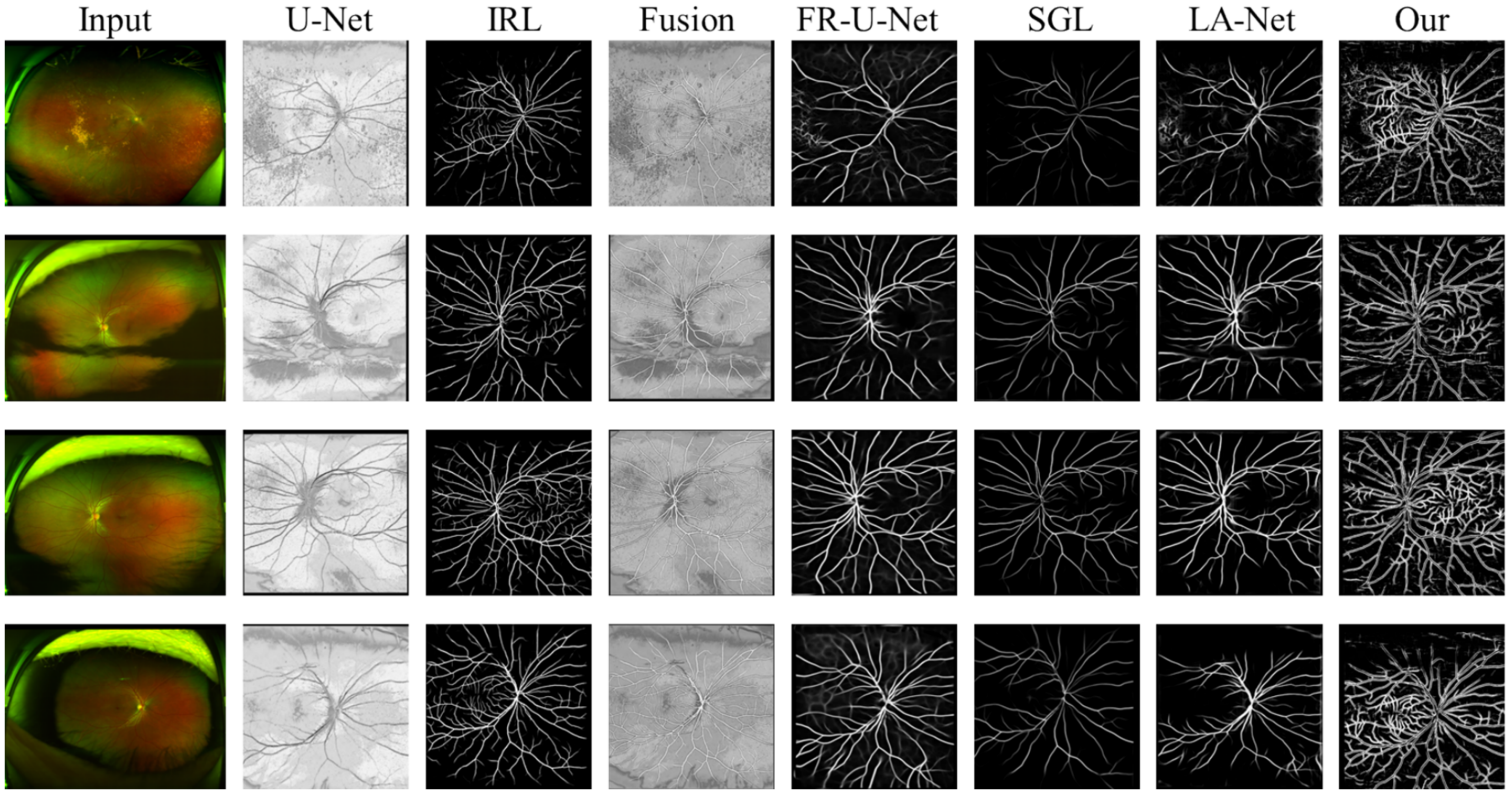
Sample images of retinal vessel segmentation performed on collected ultra-widefield retinal images using the proposed ReIU model.

Finally, in Figure 8, we present sample images of vessel segmentation performed on the collected retinal vessel images using the proposed ReIU model. Due to the absence of ground truth labels, quantitative accuracy assessment is not feasible; however, the examples demonstrate that the proposed model can adapt to some extent to the challenging task of ultra-widefield retinal vessel segmentation under suboptimal conditions.

## 5 Conclusion

With the growing prevalence of Alzheimer’s disease, there is an urgent need for a low-cost, rapid, high-precision, and non-invasive preliminary screening method. This study introduces ReIU, a model that integrates multi-source heterogeneous datasets for early AD detection, demonstrating strong performance in classifying AD-related data. Within this framework, ReIU is particularly effective at extracting retinal vessel density from fundus images, and the derived vessel density biomarkers have shown potential as training data for preliminary screening models, achieving an accuracy of 79%.

However, despite the effectiveness demonstrated in our experiments, retinal images in practical clinical settings do not exhibit distinct pathological features, and retinal vessel density does not reliably reflect AD severity. Additionally, the challenge of obtaining labels for ultra-widefield retinal images poses a significant obstacle to further progress in this subtask. In future work, we aim to address these limitations and develop more accurate and robust methods.

## Data Availability

The original contributions presented in the study are included in the article/supplementary material, further inquiries can be directed to the corresponding author/s.
